# Describing the Primary Care Actions of Oral Health Teams in Brazil

**DOI:** 10.3390/ijerph120100667

**Published:** 2015-01-12

**Authors:** Clarice Magalhães Rodrigues dos Reis, Antônio Thomaz Gonzaga da Matta-Machado, João Henrique Lara do Amaral, Marcos Azeredo Furquim Werneck, Mauro Henrique Nogueira Guimarães de Abreu

**Affiliations:** 1 Department of Community and Preventive Dentistry, Universidade Federal de Minas Gerais, Belo Horizonte, Av. Antônio Carlos, 6627 Belo Horizonte, Minas Gerais 31270.901, Brazil; E-Mails: reisclarice@gmail.com (C.M.R.R.); jhamaral1@gmail.com (J.H.L.A.); mfurquim52@gmail.com (M.A.F.W.); 2 Department of Preventive and Social Medicine, Universidade Federal de Minas Gerais, Belo Horizonte, Av. Antônio Carlos, 6627 Belo Horizonte, Minas Gerais 31270.901, Brazil; E-Mail: thomaz@nescon.medicina.ufmg.br

**Keywords:** primary health care, oral health services, evaluation

## Abstract

*Objective*: To describe the primary care actions performed by oral health teams (OHTs) that participated in a large national survey led by the Ministry of Health in 2012. *Methods*: A total of 12,403 dentists from OHTs completed a set of survey questions (response rate = 85.01%) on the organization of care, basic dental procedures and oral health surveillance actions of OHTs. Descriptive and hierarchical cluster analyses were developed. *Results*: The majority of OHTs (85.2%) reported that they performed “patient welcoming”. The delivery of services was based on a patient’s identified disease risk (83.1%), and continuity of care was ensured by 85.9% of OHTs. Individual preventive, restorative and surgical procedures were performed by the majority of the teams; however, screening for oral cancer and construction of dental prostheses/dentures occurred less frequently. Cluster 1 was composed of OHTs with the lowest proportion of oral healthcare actions related to oral cancer and dental prostheses/dentures, and the Southeastern and Southern regions had higher proportions of OHTs from cluster 2. *Conclusions*: OHTs adhere to some of the principles of primary care organizations; however, the teams perform fewer actions related to oral cancer treatment and rehabilitation with complete dentures. The geographical distribution of the clusters was unequal in Brazil.

## 1. Introduction

Since 1994, the Family Health Program (FHP) has been the main strategy used to strengthen Primary Health Care (PHC) services in the Brazilian Health System (SUS in Portuguese). In 2000, the Ministry of Health (MofH) included Oral Health Teams (OHTs) in the FHP by adding a financial incentive to encourage its implementation throughout the country. These OHTs are composed of one general dentist and one or two dental assistants [1]. The goal was to avoid models of inefficient care that excluded the adult and elderly populations in Brazil as well as models based on curativism, technicality and biologicism [2] with the aim of promoting and transitioning into models of care based on PHC principles such as accessibility, comprehensiveness, coordination, continuity, and family and community-centered care [3,4].

In 2004, the MofH launched “*Brasil Sorridente*”, the current Brazilian Oral Health Policy, as another important step toward improving the provision of oral care. The goal of this policy was to revisit the epidemiological situation found in the population survey conducted in 2003, when 20% of the population had undergone full-mouth extractions [5]. Thus, the new policy included a set of activities at the three levels of care: it increased the financial incentive for OHT implementation and improved the infrastructure of dental offices; it promoted the qualification of dentists and allied dental professionals and included dental prostheses/dentures and oral cancer screening as procedures performed by OHTs; and it established Dental Specialty Centers (CEO) and prosthetic laboratories [6].

Since then, the number of OHTs has increased around the country, and in 2013, approximately 30,000 OHTs were present in Brazil. For many municipalities, OHTs are the main and often the only means of obtaining oral health care [2]. Nonetheless, many challenges must be surpassed, including a systematic evaluation of the quality of care that is provided by OHTs. The need to focus on quality care evaluation is international [7,8]; however, there is a lack of research on oral health service evaluation worldwide.

It is important to invest in policies that assess the quality of services provided under the FHP, including the actions of OHTs. Thus, in 2011, the MofH developed a program to assess and enhance the quality of PHC in Brazil, “*Programa Nacional de Melhoria do Acesso e Qualidade da Atenção Básica* —PMAQ-AB,” which included a financial incentive [9]. The main challenge of PMAQ-AB was to stimulate a culture of evaluation among PHC professionals and managers who evaluate processes and outcomes in PHC. Additionally, PMAQ-AB offered financial incentives to the teams working in the FHP, *i.e.*, they received financial transfers based on evaluation results [10].

Given the importance of assessing oral health services in PHC and the few studies on this topic, the objective of this study was to describe the primary care actions performed by OHTs that participated in PMAQ-AB in 2012.

## 2. Methods

This descriptive study uses data from a national survey on PHC teams led by the Brazilian MofH, “*Programa Nacional de Melhoria do Acesso e Qualidade da Atenção Básica*—PMAQ-AB”. The goal of this project was to improve access to and the quality of PHC by technically and economically supporting PHC teams, which implies that each PHC team should undergo an accreditation process based on the results of an external evaluation and on the analysis of health indicators. PMAQ was based on the classical quality of care framework by Donabedian, in which quality is evaluated using structure, process and outcome parameters [9,11]. This study focused on the primary care actions of OHTs.

The interviewed population consisted of Brazilian dentists working in OHTs who completed the PMAQ-AB survey regarding oral health care. In 2012, there were 29,180 OHTs in Brazil, but MofH mandated that only 50% of those teams (*n* = 14,590) could engage in PMAQ during that year. The selection of a participating OHT was not random; health managers selected OHTs that that they would be evaluated. Of those teams, 12,403 (response rate = 85.01%) completed the survey questions. The questions were based on the principles of PHC that should be incorporated by OHTs that were part of PHC teams; these principles included access to care, health service organization, and continuity of care. The dentists were also asked about basic dental procedures (preventive, restorative/prosthetic and surgical procedures on patients of all ages) that must be performed by the OHTs according to the MofH mandate. Surveillance actions for oral cancer and for identifying the need for dental prostheses were also evaluated. In Brazil, oral health actions are developed for all ages.

The Brazilian MofH partnered with academic institutions to develop the questionnaire and to perform fieldwork, as well as to select and train interviewers who were to administer the survey throughout the country. The development of the questionnaire involved the participation of professors from five Brazilian universities and one research institution. The theoretical framework was based on the principles of PHC, and structure, process and health results were included [3,4,11]. After a pilot study, in 2012, data were collected in face-to-face interviews at the primary health care (PHC) units using a structured questionnaire. Despite not having been formally validated, the questions evaluated had an adequate internal consistency (Cronbachʼs alpha = 0.814). The dentists were volunteers in this project and could refuse to answer the questionnaire, which consisted of mostly dichotomous questions, including an option of no answer/do not know.

Three questions about access to dental care were asked: “*Is there ‘patient welcoming’ in the oral health services?*”; “*How are the appointments for oral health services scheduled?*”; “*Does the OHT use guidelines for ‘patient welcoming’?*” The organization of the health services was evaluated using the following questions: “*Does the OHT perform vulnerability assessment and disease risk classification at the first appointment?*” and “*Does the OHT offer oral health services according to patients’ identified risk?*” The questions regarding continuity of care were “*Does the OHT ensure continuity of care?*” and “*Does the OHT provide references for prosthetics services?*”. The following basic dental procedures performed by OHTs were evaluated: identification of sealants, fluoride application, amalgam filling, composite filling, dental extraction, temporary restorations, endodontic medication use in emergencies, drainage of oral abscess, supragingival scaling, root planing and coronal polishing, and oral dentures. The questions regarding oral health surveillance were “*Does the OHT have policies for identifying oral lesions and referring suspected cases of oral cancer?*”*,* “*Does the OHT register and follow suspected and confirmed cases of oral cancer?*”*,* and “*Does the OHT have policies for identifying people who need dentures?*”.

Descriptive statistical analysis was performed using proportion calculations of each variable; confidence intervals were not calculated because this was a census study. Clustering was based on six variables that evaluated oral cancer treatment and the construction of dental prostheses/dentures; the questions were as follows: *“Does the OHT provide references for prosthetics services?”, “Does the OHT perform denture impressions at the PHC dental office?”, “Does the OHT provide the dentures and follow the patients?”, “Does the OHT have policies for identifying oral lesions and referring suspected cases of oral cancer?”, “Does the OHT register and follow suspected and confirmed cases of oral cancer?”,* and *“Does the OHT have policies for identifying people who need dentures?”* Three types of clusters (including two to four clusters) were formed from the 12,402 OHTs in Brazil. The choice of two clusters was due to a better understanding of the phenomenon (PHC actions related to oral cancer and dental prostheses/dentures). The multivariate agglomerative hierarchy technique based on the furthest neighbor (CA), which is an exploratory data analysis tool for organizing observed data (in our case, OHTs) into groups (clusters) based on combinations of independent variables (in our case, PHC actions related to oral cancer and dental prostheses/dentures) and for maximizing the similarity of cases within each cluster while maximizing the dissimilarity between groups, was used for the cluster analysis. This multivariate analysis creates new groupings without any preconceived notion of what clusters may arise, and this data reduction makes it easier to manage subgroups [12]. We then compared the proportions of the two clusters to the five Brazilian geographical regions: North, Northeast, Central, Southeast and South. All analyses were performed using the Statistical Package for Social Sciences (SPSS) version 19.0. The study was submitted to and approved by the Ethics Committee for Human Research of the Universidade Federal de Minas (protocol number 31525514.9.0000.5149).

## 3. Results

Table 1 shows the results regarding access to and organization of oral health services and patients’ continuity of care in PHC facilities. The methods used for booking appointments at those health units varied, and in 38.1% of the health units, service users could book appointments on any weekday and at any time. The majority of OHTs (85.2%) reported that they performed “patient welcoming”. However, only 43.7% of those teams used guidelines for “patient welcoming”, and only 31.1% presented documents confirming this use. The offer of services was based on patient’s identified disease risk (83.1%), and continuity of care was ensured by 85.9% of the OHTs. A reference for a prosthetics service was given by 45.9% of the OHTs.

Table 2 and Table 3 show the procedures performed by OHTs. OHTs performed composite fillings (92.8%) more often than amalgam fillings, and only 33.9% of the OHTs presented documents certifying that they registered and followed patients with oral cancer (Table 3).

Actions identifying people in need of dentures were performed by 50.5% of the OHTs. However, only 9.2% of those teams performed denture impressions in PHC facilities, and this number decreased to 7.6% when certification of this action was requested. Moreover, only 10.2% of the OHTs demonstrated that they provided dentures at the PHC.

**Table 1:** Actions performed by oral health teams related to access to care, health service organization, and continuity of care in Brazil, 2012.

Variables	Frequency (%)
Is there “patient welcoming” in the oral health services?	
Yes	10,567 (85.2)
No	1823 (14.7)
Do not know/No response	13 (0.1)
How are the appointments for oral health services scheduled?	
Any weekday, at any time	4729 (38.1)
Any weekday, at specific times	1492 (12.0)
Specific days, fixed for up to three days a week	2350 (18.9)
Specific days, fixed for more than three days a week	949 (7.7)
Others	2871 (23.1)
Do not know/No response	12 (0.1)
Does the OHT use guidelines for “patient welcoming”?	
Yes, with document certification	3863 (31.1)
Yes, without document certification	1562 (12.6)
No	6965 (56.2)
Do not know/No response	13 (0.1)
Does the OHT perform vulnerability assessment and disease risk classification at the first appointment?	
Yes	11,256 (90.8)
No	1134 (9.1)
Do not know/No response	13 (0.1)
Does the OHT offer oral health services according to a patient’s identified risk?	
Yes	10,301 (83.1)
No	2089 (16.8)
Do not know/No response	13 (0.1)
Does the OHT ensure continuity of care?	
Yes, with document certification	9132 (73.6)
Yes, without document certification	1527 (12.3)
No	1744 (14.0)
*Does the OHT provide references for prosthetics services?*	
Yes	5694 (45.9)
No	6694 (54.0)
Do not know/No response	15 (0.1)

**Table 2:** Basic dental procedures performed by oral health teams in Brazil, 2012.

Variables	Frequency (%)
Sealants	
Yes	9802 (79.0)
No	2588 (20.9)
Do not know/No response	13 (0.1)
Fluoride application	
Yes	11,781 (95.0)
No	609 (4.9)
Do not know/No response	13 (0.1)
Amalgam filling	
Yes	10,881 (87.7)
No	1509 (12.2)
Do not know/No response	13 (0.1)
Composite filling	
Yes	11,511 (92.8)
No	879 (7.1)
Do not know/No response	13 (0.1)
Dental extraction	
Yes	11,524 (92.9)
No	866 (7)
Do not know/No response	13 (0.1)
Temporary restorations	
Yes	11,300 (91.1)
No	1090 (8.8)
Do not know/No response	13 (0.1)
Endodontic medication use in emergencies	
Yes	11,448 (92.3)
No	942 (7.6)
Do not know/No response	13 (0.1)
Drainage of oral abscesses	
Yes	10,230 (82.5)
No	2160 (17.4)
Do not know/No response	13 (0.1)
Supragingival scaling, root planing and coronal polishing	
Yes	11,206 (90.3)
No	1184 (9.5)
Do not know/No response	13 (0.1)
Does the OHT perform denture impressions at the PHC dental office?	
Yes, with document certification	948 (7.6)
Yes, without document certification	191 (1.5)
No	11,249 (90.8)
Do not know/No response	15 (0.1)
Does the OHT provide the dentures and follow the patients?	
Yes, with document certification	1266 (10.2)
Yes, without document certification	465 (3.7)
No	10,657 (86.0)
Do not know/No response	15 (0.1)

**Table 3:** Oral health surveillance actions performed by oral health teams in Brazil, 2012.

Variables	Frequency (%)
Does the OHT have policies for identifying oral lesions and referring suspected cases of oral cancer?	
Yes	9020 (72.7)
No	3370 (27.2)
Do not know/No response	13 (0.1)
Does the OHT register and follow suspected and confirmed cases of oral cancer?	
Yes, with document certification	4209 (33.9)
Yes, without document certification	3120 (25.2)
No	5061 (40.8)
Do not know/No response	13 (0.1)
Does the OHT have policies for identifying people who need dentures?	
Yes	6260 (50.5)
No	6128 (49.4)
Do not know/No response	15 (0.1)

Cluster 1 was composed of OHTs with the lowest proportion of oral healthcare actions related to oral cancer treatment and construction of dental prostheses/dentures (Table 4). The Southeastern and Southern regions had a higher proportion of OHTs from cluster 2 and showed the best performance of these procedures (Table 5).

**Table 4:** Primary healthcare actions related to oral cancer and dental prostheses/dentures in the two clusters in Brazil, 2012.

Variables *	Cluster 1 (*n* = 3369)	Cluster 2 (*n* = 9019)
	(Yes) %	(Yes) %
Does the OHT give references for prosthetics services?	29.5	52.1
Does the OHT perform denture impressions at the PHC dental office?	4.7	10.9
Does the OHT provide dentures and follow the patients?	6.8	16.7
Does the OHT have policies for identifying oral lesions and referring suspected cases of oral cancer?	0	100
Does the OHT register and follow suspected and confirmed cases of oral cancer?	28.6	70.6
Does the OHT have policies for identifying people who need dentures?	27.6	59.1

* Data for some variables are missing.

**Table 5:** Proportion of the two clusters according to Brazilian geographical region in 2012.

Brazilian Geographical Regions *	Cluster 1 (*n* = 3369)	Cluster 2 (*n* = 9019)
	%	%
North (*n* = 803)	40.1	59.9
Northeast (*n* = 4751)	31.2	68.8
Central (*n* = 923)	32.0	68.0
Southeast (*n* = 3929)	19.6	80.4
South (*n* = 1982)	25.0	75.0

* Data for some variables are missing.

## 4. Discussion

This study presents the primary care actions of OHTs that participated in PMAQ-AB in 2012. The results showed that OHTs adhered to some of the basic PHC principles, such as allowing access to services and comprehensive care. The teams also performed traditional clinical work, including individual preventive procedures, restorative procedures, basic periodontal procedures, extractions and emergencies. Finally, the OHTs less frequently treated oral cancer and promoted rehabilitation with complete dentures. Two clusters of OHTs performed healthcare actions related to oral cancer treatment and the construction of dental prostheses/dentures, and the distribution of these clusters differed between the different geographic regions of Brazil.

Based on the results, the majority of OHTs reported providing organized access to health services based on “patient welcoming”, as previously identified in a study performed in a large Brazilian city [13]. However, there was no sufficient use of “patient welcoming” guidelines. This result may demonstrate a certain misunderstanding regarding the meaning of welcoming service. A recent study found that the concept of “patient welcoming” is unclear to oral health care professionals, and this study showed that some professionals defined welcoming service as “qualified hearing”, while others defined it as “humanized screening” [14]. “Patient welcoming” is the act of receiving the patient in the clinic and providing a response regarding their problem. Many health services restrict care to only a limited number of health care treatments per day; people who are first in line are met first. The number of patients who can receive health care per day is limited; therefore, the complaints of many users seeking health services are not heard. This exclusive model, which is focused on disease, does not adhere to the principles of PHC. In Brazil, there is a strong demand for what is called “patient welcoming” (“Acolhimento” in Portuguese). More than a screening/triage, “patient welcoming” is the act of listening, welcoming and responding to the demands presented by subjects [14]. This approach meets the health care patient-centered requirements [15]. Another important question is how users schedule appointments for dental treatments. A large number of OHTs booked appointments on a fixed day and at a fixed time, which is considered a barrier to accessing oral health services [16].

Many teams also claimed that they perform vulnerability assessment and disease risk classification at the first appointment, but they failed to mention how those assessments were used by the teams. According to a new strategy for integrating chronic disease prevention and general health promotion, the assessment of risk and vulnerability could help OHTs provide health actions. Regarding major chronic diseases, social and environmental variables are distal causes of oral diseases. Moreover, a group of modifiable risk factors is common to many chronic diseases and injuries, as well as most oral diseases [17,18]. Therefore, there is a need to explore these issues using other available data systems and other research methodologies to better understand the use of risk and vulnerability in the practice of OHTs.

The results of this study suggest that OHTs have been performing basic oral health procedures, and these results could indicate that the infrastructure of dental offices in the FHP is adequate. This could be explained by the large financial investments over the last ten years due to the National Oral Health Policy (“*Brasil Sorridente*”), which aimed to restructure oral health services in the FHP [1,16]. It is important to highlight that the Alma-Ata Declaration has influenced PHC organization in Brazil and in other countries and could also explain the high number of basic oral health procedures that have been performed [19]. However, the evaluation of infrastructure alone is not adequate for determining the quality of a health service; evaluating the perspective of the patient is also very important [11].

This study also showed that OHTs are not performing procedures for the early detection of oral cancer. This result represents an important warning for OHTs and FHP managers because oral cancer has a high mortality rate and because early diagnosis is crucial for patient survival [20]. In this sense, due to its structure, the FHP is an excellent resource for the early detection and tracking of cases of oral cancer. Community agents have direct access to patient residences, which should help in the early detection and monitoring of cases. Oral cancer screening during vaccination campaigns for the elderly was identified as an efficient action in Brazil [21]. Furthermore, lack of knowledge on oral cancer prevention and a lack of undergraduate courses on this topic could be associated with these practices [22,23,24,25,26].

Another important finding was that dentures and prostheses were rarely offered. There are no guidelines in the MofH mandating that OHTs provide dentures or other dental prostheses. However, removable dental prostheses were sometimes included in primary care as a strategy to encourage their use and to increase the list of services. Thus, the decision to provide prostheses depends on the OHT. If the OHT decides to provide prostheses, they must have access to a lab (*i.e.*, their own lab or an outside lab) that can fabricate them. In this case, one or more dentists will be assigned to conduct all proceedings related to removable dental prostheses. “Making dentures” was recently included in public oral health procedures, which is an important step forward that needs to be extended given the epidemiological situation of the edentulous population in Brazil [5,27].

There were inequalities in health service organization among the different geographical locations of the clusters. These regions present clear differences in socioeconomics and demographics, and there are important socioeconomic inequalities among population groups and regions. The more developed regions are the Southeastern and Southern regions [28]. Because a health service organization could be influenced by socio-economic and demographic variables [29,30,31], it is not surprising that that these regions had higher proportions of OHTs from cluster 2. Moreover, if OHTs do not provide dental prostheses/dentures or treatment for oral cancer, the reasons for this should be identified in other studies.

This study showed that OHTs are not completely meeting the demands of the population, especially the demands of the adult and elderly populations, in Brazil. The epidemiology of the edentulous community in these age groups and the high frequency of oral cancer among the elderly have not been adequately addressed by OHTs. The most recent Brazilian oral epidemiological survey showed that 22.4% of adults had fewer than 21 natural teeth and that 53.7% of the elderly were edentulous [27,32].

One limitation of a descriptive cross-sectional study is the low analytical power of the results. Additionally, in the first evaluation of PMAQ-AB performed in 2012, the MofH fixed the adherence to OHTs at 50% in each municipality. This fixed percentage may have created a selection bias in this study because the most well-structured teams might have joined the program first.

This study is innovative because it uses a large national dataset to which approximately 50% of the OHTs contributed. Thus far, this is the most comprehensive evaluation of oral health conducted in Brazil, and according to our literature review, no other country has conducted an oral health care study of this size. PMAQ-AB will be held biannually and will provide longitudinal data, which demonstrates the importance of this study as a baseline for monitoring oral health care in Brazil. Currently, the FHP is an established policy for implementing PHC in Brazil, and the numbers of OHTs have increased in recent years. Describing the PHC actions of OHTs through their participation in a large national survey is an important baseline from which to further explore the care provided to citizens.

## 5. Conclusions

OHTs adhere to some of the principles of primary care organizations. However, the teams perform fewer actions related to oral cancer treatment and rehabilitation with complete dentures. Furthermore, the geographical distributions of the clusters were unequal.

## References

[1] Junqueira S.R., Pannuti C.M., Sde M.R. (2008). Oral health in Brazil—Part I: Public oral health policies. Braz. Oral Res..

[2] Souza T.M.S., Roncalli A.G. (2007). Saúde bucal no Programa Saúde da Família : Uma avaliação do modelo assistencial. Cad. Saude Publica.

[3] Starfield B. (1992). Primary Care: Concept, Evaluation and Policy.

[4] Starfield B. (2009). Toward international primary care reform. Can. Med. Assoc. J..

[5] Peres M.A., Barbato P.R., Reis S.C.G.B., Freitas C.H.S., Antunes J.L.F. (2013). Perdas dentárias no Brasil: Análise da Pesquisa Nacional de Saúde Bucal 2010. Rev. Saúde Pública.

[6] Pucca-Junior G.A., Costa J.F.R., Chagas L., Sivestre R.M. (2009). Oral health policies in Brazil. Braz. Oral Res..

[7] World Health Organization (WHO) (2009). Monitoring and Evaluation of Health Systems Strengthening: An Operational Framework.

[8] Alberta Government (2013). Primary Health Care Evaluation Framework.

[9] Voinea-Griffin A., Fellows J.L., Rindal D.B., Barasch A., Gilbert G.H., Safford M.M. (2010). Pay for performance: Will dentistry follow?. BMC Oral Health.

[10] (2012). Brasil Saúde Mais Perto de Você—Acesso e Qualidade—Programa Nacional de Melhoria do Acesso e da Qualidade da Atenção Básica (PMAQ)—Manual Instrutivo.

[11] Donabedian A. (1966). Evaluating the quality of medical care. Milbank Mem. Fund Quart..

[12] Johnson R.A., Wichem D.W. (2007). Applied Multivariate Statistical Analysis 6.

[13] Mattos G.C.M., Sirineu C.G., Teixeira B.R., Gallagher J.E., Paiva S.M., Abreu M.H.N.G. (2014). A survey of the perception of comprehensiveness among dentists in a large Brazilian City. Int. J. Environ. Res. Public Heal..

[14] Mitre S.M., Andrade E.I.G., Cotta R.M.M. (2012). Avanços e desafios do acolhimento na operacionalização e qualificação do Sistema Único de Saúde na Atenção Primária: Um resgate da produção bibliográfica do Brasil. Cienc Saude Colet.

[15] Dickinson W.P., Miller B.F. (2010). Comprehensiveness and continuity of care and the inseparability of mental and behavioral health from the patient-centered medical home. Fam. Syst. Health.

[16] Costa J., Chagas L., Silvestre R. (2006). A política nacional de saúde bucal do Brasil: Registro de uma conquista histórica. http://bvsms.saude.gov.br/bvs/publicacoes/Sala5545.pdf..

[17] Riley J.L., Qvist V., Fellows J.L., Rindal D.B., Richman J.S., Gilbert G.H., Gordan V.V., DPBRN Collaborative Group (2010). Dentistsʼ use of caries risk assessment in children: Findings from the dental practice-based research network. Gen. Dent..

[18] Petersen P.E. (2008). World Health Organization global policy for improvement of oral health—World Health Assembly 2007. Int. Dent. J..

[19] Jatrana S., Crampton P., Filoche S. (2009). The case for integrating oral health into primary health care. N. Z. Med. J..

[20] Van der Waal I. (2013). Are we able to reduce the mortality and morbidity of oral cancer; Some considerations. Med. Oral Patol. Oral Cir. Bucal.

[21] Almeida F.C., Cazal C., Pucca Júnior G.A., Silva D.P., Frias A.C., Araújo M.E. (2012). Reorganization of secondary and tertiary health care levels: Impact on the outcomes of oral cancer screening in the São Paulo State, Brazil. Braz. Dent. J..

[22] Horowitz A.M., Siriphant P., Sheikh A., Child W.L. (2001). Perspectives of Maryland dentists on oral cancer. J. Am. Dent. Assoc..

[23] Warnakulasuriya K.A., Johnson N.W. (1999). Dentists and oral cancer prevention in the UK: Opinions, attitudes and practices to screening for mucosal lesions and to counselling patients on tobacco and alcohol use: Baseline data from 1991. Oral Dis..

[24] Decuseara G., MacCarthy D., Menezes G. (2011). Oral cancer: Knowledge, practices and opinions of dentists in Ireland. J. Ir. Dent. Assoc..

[25] Razavi S.M., Zolfaghari B., Foroohandeh M., Doost M.E., Tahani B. (2013). Dentistsʼ knowledge, attitude, and practice regarding oral cancer in Iran. J. Cancer Educ..

[26] Rahman B., Hawas N., Rahman M.M., Rabah A.F., Al Kawas S. (2013). Assessing dental studentsʼ knowledge of oral cancer in the United Arab Emirates. Int. Dent. J..

[27] (2011). Projeto SBBrasil 2010: Pesquisa Nacional de Saúde Bucal—Resultados Principais.

[28] Pan American Health Organization (2007). Technical Cooperation Strategy for PAHO/WHO and the Federative Republic of Brazil, 2008–2012.

[29] Andersen R.M. (1995). Revisiting the behavioral model and access to medical care: Does it matter?. J. Health Soc. Behav..

[30] Travassos C., Martins M. (2004). A review of concepts in health services access and utilization. Cad Saude Publica..

[31] Esteves R.S., Mambrini J.V., Oliveira A.C., Abreu M.H. (2013). Performance of primary dental care services: An ecological study in a large Brazilian city. Sci. World J..

[32] Peres M.A., Barbato P.R., Reis S.C.G.B., Freitas C.H.S.M., Antunes J.L.F. (2013). Tooth loss in Brazil: Analysis of the 2010 Brazilian oral health survey. Rev. Saude Publica.

